# Western Pennsylvania Veterinary Medical Association

**Published:** 1891-09

**Authors:** 


*Western Pennsylvania Veterinary Medical Association.*—A number of graduate veterinarians of Pittsburgh and vicinity, met several times at the office of Dr. J. C. McNeil, No. 26 Fourth St., and finally on July 24th organized the above named association, and elected the following named officers to serve for one year, viz:

President, E. J. Carter, V. S.; Vice-President, H. F. Doris, D. V. S.; Secretary, James A. Waugh, V. S.; Treasurer, James C. McNeil, V. M. D.; Directors, H. S. Richards, V.S. Charles Falconer, V.S. and C. Z. Laberge, V. M. Monthly meetings will be held regularly on the first Saturday of every month.

James A. Waugh has promised to read at the next meeting a paper entitled "Electrical Accidents to Domestic Animals."

JAMES A. WAUGH, V.S., *Secretary.*

*Veterinary Medical Association of New Jersey.*—The 23d regular meeting of the New Jersey Veterinary Medical Association, was held in Saenger Hall, Newark, New Jersey; on Tuesday August 13, 1891. The meeting was called at 12 o'clock (noon). by the President, Dr. R.R. Letts. The Secretary called the roll, and the following members were present:

Drs. W. B. E. Miller, Camden; R. R. Letts, Hoboken; J. W. Hawk, Newark; J. C. Dustan, Morristown; S. Lockwood, Woodbridge; W. H. Cooper, Jersey City; L. P. Hurley, Hopewell; B. F. King, Little Silver; J. Kehoe, Lyndhurst; H. W. Rowland, Newark; W. Runge, Newark; R. O. Hasbunck, Passaic; M. M. Stage, Dover; J. Gerth, Jr., Newark.

The minutes of the last meeting were read and approved. President Letts made a short address. The Secretary and the Treasurer read their reports, and the following applications for membership were accepted:

Drs. Eugene Britten, Long Branch City; Hugh Exton, Washington; E. W. Schuppan, Jersey City; F. Lippencott, Swedeboro.

Dr. Miller reported for the Board of Censors, and recommended Exton, Britten, and Schuppan, who were elected members. Miscellaneous business was then taken up.

A number of letters were read from veterinarians, and from veterinary associations. An able address was made by Dr. W. H. Hoskins, of Philadelphia.

Camden was selected as the place for the next meeting.

W. H. Cooper, *Secretary.*

